# The use of appropriate, inappropriate and no comparators in efficacy trials for European Medicines Agency-approved drugs 2011–2024: a retrospective cohort study

**DOI:** 10.1016/j.eclinm.2026.104319

**Published:** 2026-11-09

**Authors:** Camille E.G. Glaus, Cascal Herrle, Simon Hediger, Kerstin Noelle Vokinger

**Affiliations:** a Academic Chair for Regulation in Law, Medicine, and Technology, Faculty of Law, University of Zurich, Zurich, Switzerland; b Academic Chair for Regulation in Law, Medicine, and Technology, Department of Health Sciences and Technology, ETH Zurich, Zurich, Switzerland

**Keywords:** Drugs, Clinical trials, Comparative clinical evidence

## Abstract

**Background:**

New drugs should ideally have an added therapeutic benefit compared to existing treatments. In order to determine whether comparative evidence was available at approval, we analyzed the proportion of new drugs that were tested in efficacy trials based on appropriate, inappropriate and no comparators overall, over time and when differentiating between therapeutic areas.

**Methods:**

In this retrospective cohort study, we identified all new drugs approved by the EMA January 1, 2011 and December 31, 2024. The Federal Joint Committee determines the appropriate comparator of new drugs launched in Germany in publicly available assessment reports. We used these reports to determine whether the efficacy trials for each population of the drugs were conducted with the appropriate comparator. A drug indication was qualified as having compared with the appropriate comparator if at least one of its populations had an appropriate comparator. To analyze whether there was a statistically significant trend over time we estimated separate logistic regression models.

**Findings:**

We included 321 drug indications with 649 populations. 186/321 (58%) had at least one population tested with an appropriate comparator. Among the remaining indications, 87/321 (27%) had at least one population tested with an inappropriate comparator, and of those indications with at least one population not tested with an appropriate or inappropriate comparator, 48/321 (15%) had at least one population approved based on a single-arm trial. Differences occurred across therapeutic areas. Drug indications approved in 2023–2024 had 76% lower estimated odds of at least one population with an appropriate comparator compared with 2011–2012 (p-value: 0.01). By contrast, those approved in 2023–2024 had 7.5 times higher odds (p-value: 0.04) compared with 2011–2012 of being tested based on a single-arm trial.

**Interpretation:**

Less than two-thirds of drug indications were tested with an appropriate comparator as determined by the Federal Joint Committee, with a significant decrease over the past decade and a significant increase of single-arm trials. Other institutions may define the appropriate comparator differently. Future research is needed to assess the impact of the comparator choice on pricing and reimbursement.

**Funding:**

Swiss National Science Foundation.

**Research in context**

**Evidence before this study**

A search of the current literature was conducted August 13, 2026 using PubMed for studies in English, using the keywords “appropriate comparator”, “comparative efficacy”, “clinical trial”, and “drugs”. Studies showed that not all new drugs had one or more controlled trials with an active comparator at the time of approval or assessed the quality of the control arms in randomized trials based on international guidelines for drugs indicated for specific diseases. However, none has quantified comprehensively overall, across therapeutic areas and over time the proportion of new drugs that were based on efficacy trials with appropriate comparators, inappropriate comparators, and no comparator drugs (single-arm).

**Added value of this study**

Using the information of the Federal Joint Committee, less than two-thirds of new drugs had at least one population which was tested with an appropriate comparator, with a significant decrease between 2011-2012 and 2023–2024, as well as differences across therapeutic areas. Among the remaining drugs, 27% of the drugs had at least one population tested with an inappropriate comparator, and of the further remaining drugs, 15% had at least one population approved based on a single-arm trial, with the proportion of latter showing a significant increase between 2011-2012 and 2023–2024.

**Implications of all the available evidence**

Clinical trials with appropriate comparators should be more routinely used for new drug approvals. Second, drug labels could include information whether trials with appropriate comparators were conducted. This would guide physicians and patients in their decision-making. Third, reimbursement bodies could take evidence gaps due to lack of trials with appropriate comparators into account in their price negotiations. Future research is needed to understand whether the choice of an appropriate comparator is reflected in reimbursement and pricing.

## Introduction

New drugs should ideally have an added therapeutic benefit compared to existing treatments.^1^ To determine whether a new drug is superior, equivalent, or inferior to available alternatives, comparative efficacy evidence is indicated.^2^ Preferably, the benefit of a new drug is tested with the appropriate comparator.^1^, ^3^ The choice of the appropriate comparator may vary depending on the country. Such information is important to inform patients and clinicians and support them in their decision making.^2^, ^3^ Also reimbursement bodies in different countries, for example, Germany, France, Switzerland or Canada, rely on such information as a basis for reimbursement decisions and price negotiations.^1^, ^3^ In Germany, the Federal Joint Committee (Gemeinsamer Bundesausschuss, G-BA) determines the appropriate comparator of new drugs (including one or more targeted populations within a drug).^1^ All of this information is publicly available, and is also used by scientists as well as other countries, for example, Switzerland.^4^, ^5^

Lack of information on benefit of new drugs compared to existing appropriate treatments results in important evidence gaps when these new drugs are introduced into the market.^2^ It may bias a trial in favor of the experimental arm and reduces a trial’s external validity since the clinical question of whether the new drug is superior to prevailing practice cannot be answered.^6^ As a consequence, patients’ ability to make informed treatment decisions consonant with their preferences might be compromised.^1^, ^2^ Lack of information also harms healthcare systems.^1^ The evidence gap may jeopardize quality care and impede decision making, particularly on highly priced drugs in economically strained situations.^1^

Prior studies demonstrated that not all new drugs approved in Europe and the US had one or more controlled trials with an active comparator at the time of approval.^7^, ^8^ Previous studies also assessed the quality of the control arms in randomized clinical trials according to international guidelines for drugs indicated for specific diseases.^6^, ^9^, ^10^, ^11^ To systematically understand and quantify the evidence gap overall and over time, we analyzed the proportion of new drugs approved between 2011 and 2024 that were based on efficacy trials with appropriate comparators, inappropriate comparators, and no comparator drugs (single-arm). We also differentiated between the therapeutic areas and assessed trends over time. To determine whether the appropriate comparator was selected, we used the granular and public information from the Federal Joint Committee.

## Methods

### Data sources and extraction

We used publicly available data of the European Medicines Agency (EMA) to identify all new drugs (excluding diagnostic agents) approved between 1 January 2011 and 31 December 2024.^12^ For each drug, we collected the approval pathway, indication and approval date. We only included first indications. If multiple indications were approved as first indications, we included all of them in our study cohort. Drug indications were categorized in therapeutic areas based on the WHO’s Anatomical Therapeutic Classification system.^13^

During the health technology assessment (HTA) assessment, the Federal Joint Committee identifies for each EMA-approved drug indication the appropriate comparator (“zweckmässige Vergleichstherapie”), which is the established standard therapy (approved and reimbursed in Germany) with which the populations of the new drug indication should be compared.^14^, ^15^ In cases of more than one target population (e.g., mutation, age, line of treatment; see also eTable 1 for specific examples) the appropriate comparator is defined for each of these populations.^14^, ^15^ Populations are determined by the Federal Joint Committee and are based on the EMA-approved indications.^16^ Thus, all populations correspond to EMA-approved indications.^16^ The Federal Joint Committee determines the appropriate comparator in accordance with international standards of evidence-based medicine.^16^ The appropriate comparator is generally a drug authorized and reimbursed in Germany.^16^, ^17^ An off-label use may be considered if it is widely used in clinical practice.^16^, ^17^ If appropriate, watchful waiting or best supportive care can also be qualified as the appropriate comparator.^1^, ^16^ The Federal Joint Committee can also consider more than one treatment as the appropriate comparator. In such cases, they list all the appropriate comparators. The Institute for Quality and Efficiency in Healthcare (Institut für Qualität und Wirtschaftlichkeit im Gesundheitswesen, IQWiG), which assesses the therapeutic value of new drugs relevant for price negotiation between the Federal Joint Committee and the manufacturer, systematically assesses for each population of a drug whether the selected comparator was appropriate. We excluded drug indications for which the Federal Joint Committee did not determine an appropriate comparator. This occurred for several reasons: the sponsor did not submit any data for the HTA evaluation of the drug indication, the HTA evaluation was still ongoing or not publicly available, the drug indication was exempt from the determination of an appropriate comparator because it concerned a reserve antibiotic without an HTA assessment or the drug indication was an orphan drug defined as such by the EMA (see also Fig. 1).^14^ To qualify for orphan designation by the EMA, a drug indication must be intended for the treatment, prevention or diagnosis of a disease that is life-threatening or chronically debilitating, the prevalence of the condition in the EU must not be more than 5 in 10,000 or it must be unlikely that marketing of the drug indication would generate sufficient returns to justify the investment needed for its development, and no satisfactory method of diagnosis, prevention or treatment of the condition concerned can be approved, or, if such a method exists, the drug indication must be of significant benefit to those affected by the condition.^18^ However, if the annual revenues of an orphan drug indication exceed EUR 30 million (EUR 50 million until November 2022) or if it loses its orphan status because the conditions are no longer fulfilled, the Federal Joint Committee and Institute for Quality and Efficacy in Healthcare conduct a health technology assessment and determine the appropriate comparator.^19^, ^20^, ^21^ In latter cases, we were able to include the orphan drug indications in our study cohort.

**Fig. 1:**
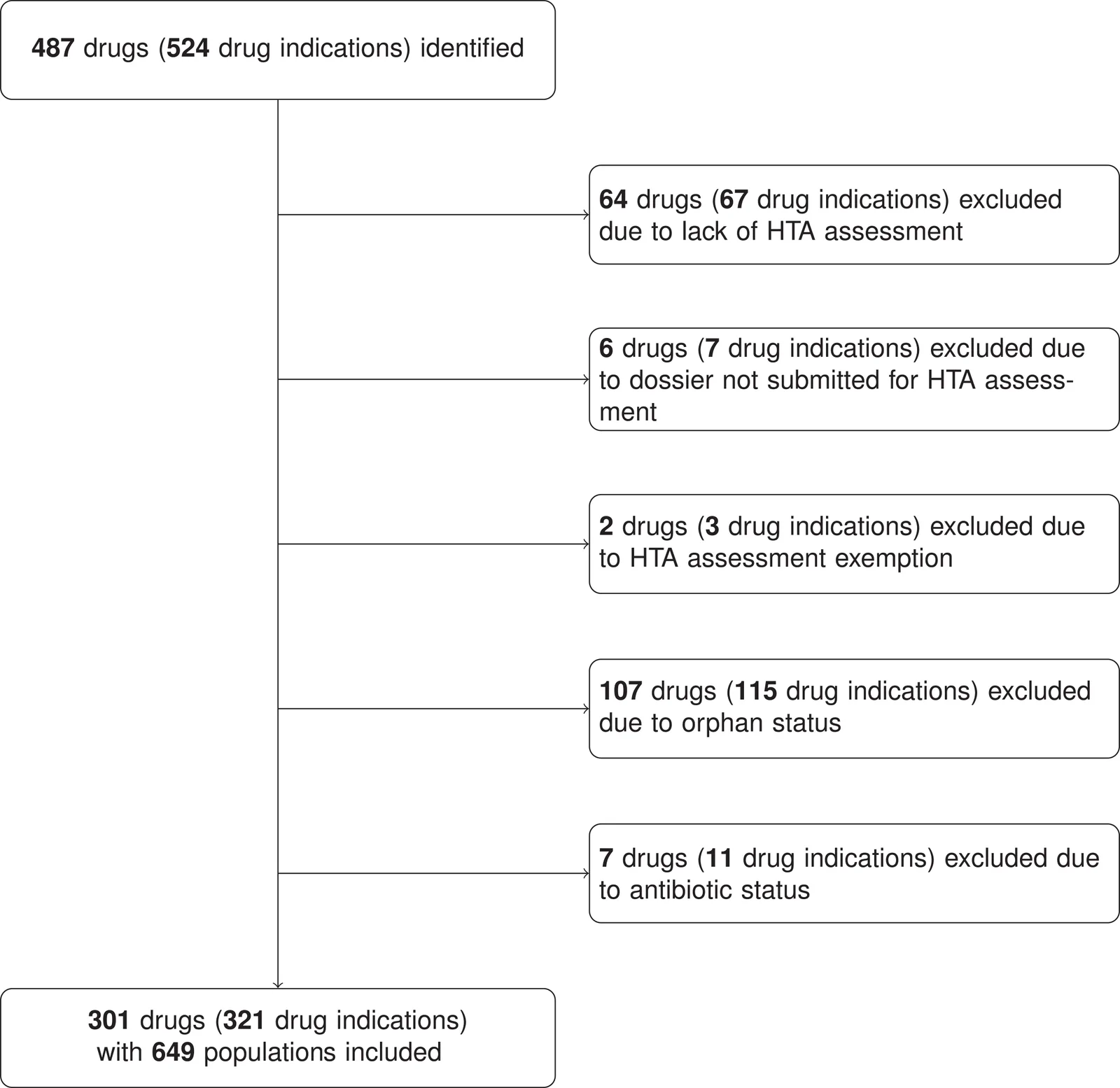
Study profile.

We used the publicly available assessment reports of the Federal Joint Committee and Institute for Quality and Efficiency in Healthcare to determine whether the efficacy trials for each population of the drug indications in our study cohort were conducted with the appropriate comparator, inappropriate comparator or with no comparator (single-arm).^22^ These categories were mutually exclusive. The comparator was considered inappropriate if the treatment administered in the control arm was not listed as an appropriate comparator by the Federal Joint Committee for the given population. When best supportive care was the designated appropriate comparator, we relied on the published assessments of the Federal Joint Committee and Institute for Quality and Efficiency in Healthcare to determine whether the trial’s control arm was appropriate. The correct use of the comparator (e.g., correct administration) was not evaluated. The included efficacy trials consisted of the studies submitted to the Federal Joint Committee and Institute for Quality and Efficiency in Health Care. These were the same efficacy studies (pivotal and supportive) submitted to the EMA for marketing authorization. In some cases, additional trials were also included if new data had become available in the interim period between the EMA submission and the Federal Joint Committee assessment. If no patients from a given population were represented in any of the submitted data based on the assessment reports, we defined this category as “no data available.” The appropriate comparator may evolve over time: Our analysis was conducted based on the evaluation report of the Federal Joint Committee at the time of the evaluation. If more than one trial was conducted for a population, we included the trial which was considered as having selected the most adequate comparator treatment. Examples are outlined in eTable 1. We considered the public reports of the Federal Joint Committee until 31 October 2025.

### Statistical analysis

We analyzed for each population of the included drug indications in our study cohort, which of the four categories were fulfilled (appropriate comparator, inappropriate comparator, single-arm, no data available). In our main analysis, a drug indication was qualified as having compared with the appropriate comparator if at least one of its populations had an appropriate comparator. If none of the populations of a drug indication had an appropriate comparator, but at least one population had an inappropriate comparator, we classified the drug indications as having compared with an inappropriate comparator. If none of the populations had an appropriate or inappropriate comparator, but at least one population was tested based on a single-arm trial, we qualified the drug indication as tested based on a single-arm trial. If a drug indication did not fulfill any of these three categories and if the manufacturer did not submit any data to the Federal Joint Committee and Institute for Quality and Efficiency in Healthcare, we classified the drug indication as “no data available.” In our sensitivity analysis, we qualified an approved drug indication in a category (appropriate comparator, inappropriate comparator, single-arm, no data available) if it was fulfilled at least by one population of an indication. Thus, for the sensitivity analysis it was possible that a drug indication was counted in more than one category (for example, if a drug indication had two populations, one was tested with an appropriate comparator and the other with an inappropriate comparator, we assigned the drug indication to both categories).

To analyze whether there was a statistically significant trend over time in the number of drug indications with at least one population using (1) an appropriate comparator, (2) an inappropriate comparator, or (3) a single-arm design, we estimated separate Firth’s logistic regression models. In each model, the binary outcome was defined as the given category versus its complement (i.e., all other indications). This outcome was regressed on the EMA approval period, controlling for therapeutic area and approval pathway (standard versus expedited). For the therapeutic area the reference category was cancer and for the approval pathway it was expedited pathway.

We conducted additional analyses to compare drug indications approved through an expedited pathway (conditional marketing authorization or accelerated assessment) versus those approved through the standard pathway. We qualified conditional marketing authorization and accelerated assessment as expedited pathways. Specifically, we compared the proportion of drug indications for which at least one population used the appropriate comparator between those approved through the conditional marketing authorization pathway and those approved via the standard approval pathway. We conducted an analogous comparison for the proportion of drug indications for which at least one population was based on a single-arm trial. We conducted the same analyses for drug indications approved through the accelerated assessment pathway and standard approval pathway. For each of the four estimated differences, we reported classical 95% confidence intervals using the t.test function from the stats R package.

All statistical analyses were prespecified and conducted in R (version 4.5.1).

### Ethics

The study was exempted from ethical approval because it used nonidentifiable data and did not include human participants.

### Role of the funding source

The funders had no involvement in the study design, data collection, data analyses, data interpretation, or the writing of the study.

## Results

We identified 487 drugs (524 drug indications) that were approved between 2011 and 2024 by the EMA. 107 orphan drugs (115 drug indications), seven reserve antibiotic drugs (11 drug indications) were excluded because they lacked an assessment, for 64 drugs (67 drug indications) no assessment was conducted, for six drugs (seven drug indications) the manufacturer did not submit the necessary information, and two drugs (three drug indications) were exempted from assessment. For these drugs the appropriate comparator was not determined and we excluded them. Thus, we included 301 drugs (321 drug indications) with 649 populations in our study cohort (Fig. 1). Of these, 44 drug indications were granted conditional marketing authorization, and 27 accelerated assessment. Our study cohort included 39 orphan drugs (42 drug indications) with 63 populations.

Drug indications for cancer treatment represented the largest therapeutic area of the 321 drug indications with 123 drug indications (38%), followed by 44 (14%) blood and cardiovascular drug indications, and 38 (12%) immunomodulators (Table 1).

**Table 1:** Overview number of drugs, drug indications and populations.

Therapeutic area	Number of drugs	Number of drug indications	Number of populations
All	301	321	649
Alimentary and metabolism	28 (9%)	28 (9%)	85 (13%)
Anti-infectives	32 (11%)	33 (10%)	127 (20%)
Blood and cardiovascular system	41 (14%)	44 (14%)	56 (9%)
Cancer	109 (36%)	123 (38%)	220 (34%)
Dermatologicals	7 (2%)	7 (2%)	7 (1%)
Genito-urinary system and hormones	8 (3%)	8 (2%)	9 (1%)
Immunomodulators	38 (13%)	38 (12%)	76 (12%)
Nervous and musculo-skeletal system	25 (8%)	27 (8%)	48 (7%)
Respiratory system	9 (3%)	8 (2%)	14 (2%)
Sensory organs	4 (1%)	5 (2%)	7 (1%)

The number of populations for a drug indication varied between one and 13, with a median of two populations (IQR: [1,2]). Best supportive care was designated as the appropriate comparator for 71/649 (11%) populations and watchful waiting for 18/649 (3%) populations.

For 116/321 (36%) drug indications, all populations were tested with the appropriate comparators, for 82/321 (26%), trials for all populations were conducted with inappropriate comparators, and for 44/321 (14%) all populations were tested based on single-arm studies.

Of the 321 drug indications, 186 (58%) had at least one population which was tested with an appropriate comparator. Among the remaining drug indications with no population tested with an appropriate comparator, 87/321 (27%) were tested based on at least one population with an inappropriate comparator, and of those drug indications with no population tested with an appropriate or inappropriate comparator, 48/321 (15%) were tested based on a single-arm trial, and for 0/321 (0%) no data was available (Fig. 2).

**Fig. 2:**
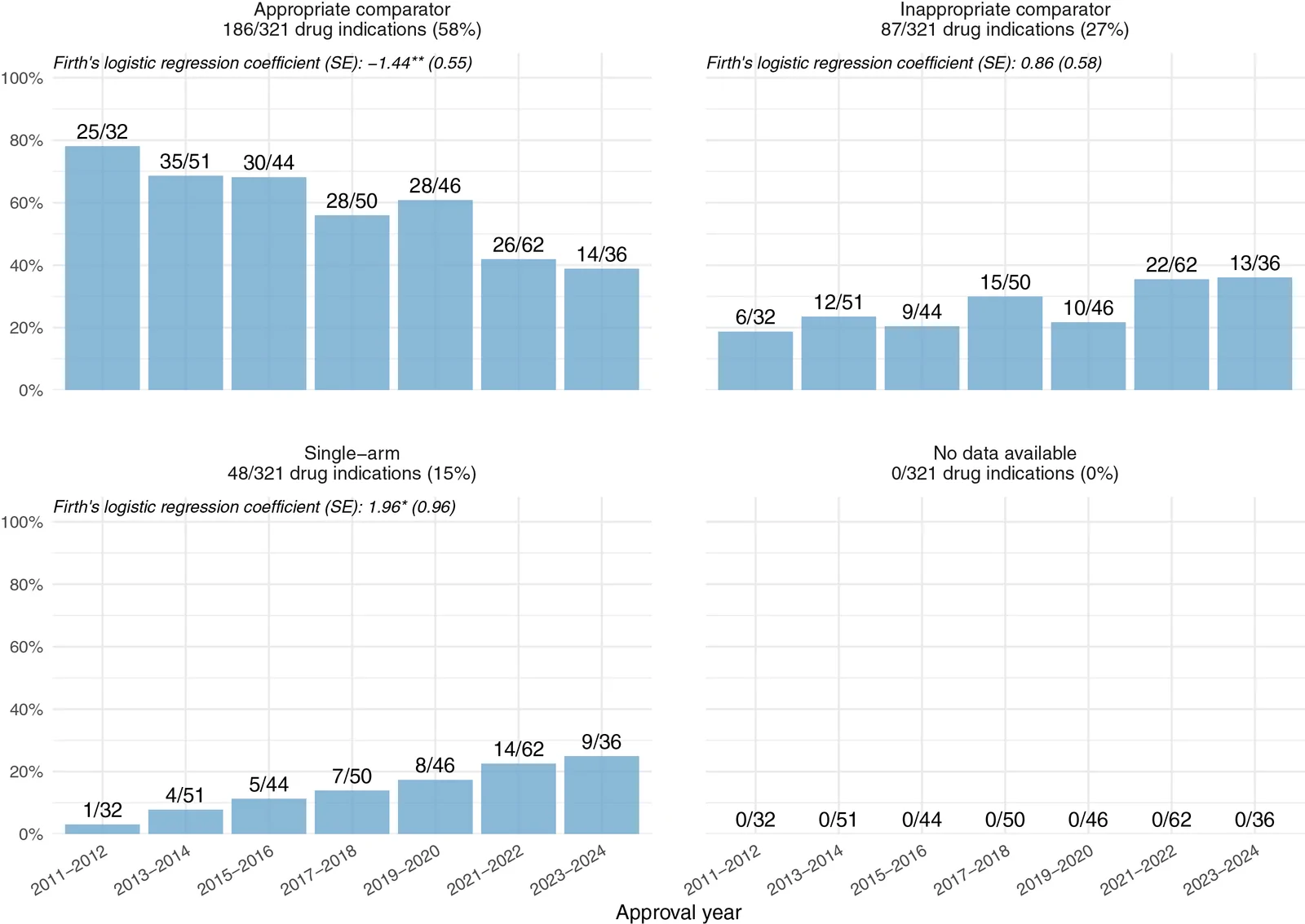
Comparators of drug indications overall and over time. *Notes:* A drug indication was classified as having compared with an appropriate comparator if at least one population within a drug indication had an appropriate comparator. If none had an appropriate comparator, but at least one population had an inappropriate comparator, the drug indication was classified as having compared with an inappropriate comparator. If none of the populations had an appropriate or inappropriate comparator, but at least one was based on a single-arm trial, the drug indication was classified in the single-arm category. If no data was submitted by the manufacturer, it was classified as “no data available”.

For the sensitivity analysis, the results were similar, i.e., most drug indications had at least one population (186/321 drug indications, 58%) which was tested with an appropriate comparator, followed by those with an inappropriate comparator (135/321 drug indications, 42%), 59/321 (18%) with no comparator, and for 31/321 (10%) no data was available (eFig. 1). Also when focusing only on orphan drug designation, most drug indications (21/42, 50%) had at least one population that used an appropriate comparator.

Differences were observed between the therapeutic areas and between drug indications. While all drug indications targeting the respiratory system and sensory organs had at least one population which was tested with an appropriate comparator, 76% (25/33) of the anti-infective drug indications had at least one population which was tested with an appropriate comparator, 66% (25/38) of the immunomodulatory drug indications, 62% (5/8) of the genito-urinary and hormones drug indications, 52% (64/123) of the cancer drug indications, 45% (20/44) of the drug indications targeting blood and cardiovascular system, 44% (12/27) of the drug indications targeting the nervous and muscle system, and 29% (2/7) dermatological drug indications. Differences were also observed between the drug indications within a therapeutic area. While all eight drugs indicated for treatment of prostate cancer had at least one population that used an appropriate comparator, only seven out of 20 drugs indicated for treatment of non-small cell lung cancer and only one out of 14 drugs indicated for lymphoma had at least one population that used an appropriate comparator (Fig. 3).

**Fig. 3:**
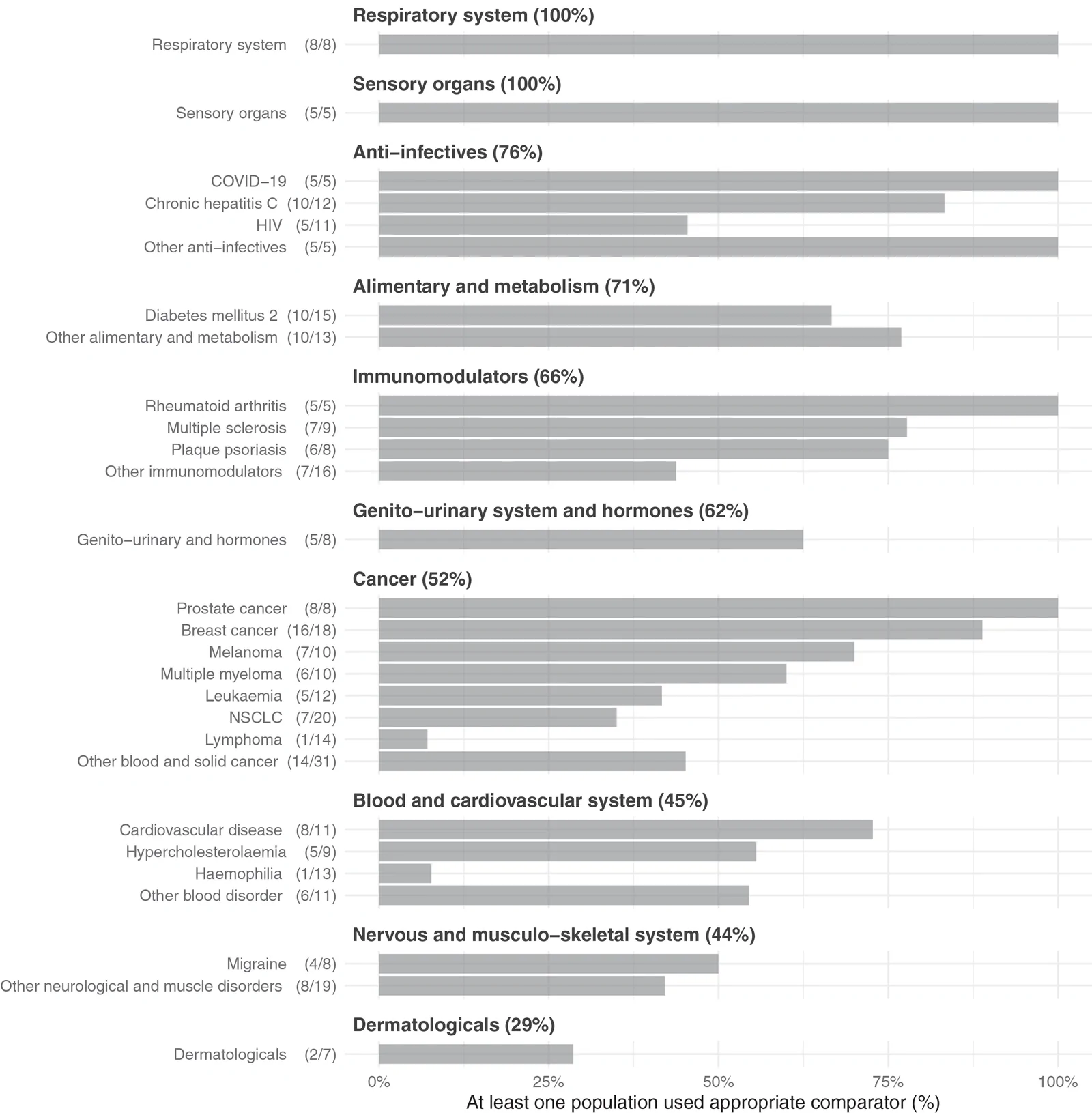
Comparator drugs across therapeutic areas (2011–2024). *Notes:* A drug indication was counted as using the appropriate comparator if at least one population within a drug indication was tested using the appropriate comparator.

Between 2011 and 2024, the proportion of drug indications with at least one population tested with an appropriate comparator decreased from 25/32 (78%) for drug indications approved 2011–2012 to 14/36 (39%) for drug indications approved 2023–2024. When controlling for the therapeutic area, the logistic regression estimated that drug indications approved in 2023–2024 had 76% lower odds of having at least one population tested with an appropriate comparator compared with those approved in 2011–2012 (p-value: 0.01) (Fig. 2, Table 2).

**Table 2:** Trends over time (regression analysis).

Coefficient	Appropriate comparator	Inappropriate comparator	Single-arm
	Estimate (SE; p-value)	Estimate (SE; p-value)	Estimate (SE; p-value)
(Intercept)	0.53 (0.48; 0.26)	−2.77⁎⁎ (0.59; 0.00)	−1.10 (0.84; 0.19)
EMA authorization period:			
2013–2014	−0.48 (0.53; 0.37)	0.35 (0.56; 0.53)	0.82 (1; 0.43)
2015–2016	−0.32 (0.54; 0.56)	0.26 (0.59; 0.66)	0.70 (0.97; 0.49)
2017–2018	−0.94^†^ (0.51; 0.07)	0.63 (0.55; 0.25)	1.50 (0.95; 0.12)
2019–2020	−0.61 (0.53; 0.25)	0.17 (0.58; 0.77)	1.49 (0.95; 0.12)
2021–2022	−1.46⁎⁎ (0.5; 0.00)	0.98^†^ (0.53; 0.06)	1.74^†^ (0.91; 0.05)
2023–2024	−1.44⁎⁎ (0.55; 0.01)	0.86 (0.58; 0.13)	1.96⁎ (0.96; 0.04)
Therapeutic area:			
Alimentary and metabolism	0.41 (0.47; 0.38)	0.26 (0.48; 0.60)	−1.98^†^ (1.39; 0.07)
Anti-infectives	0.97⁎ (0.45; 0.02)	0.06 (0.48; 0.91)	−2.21⁎⁎ (0.87; 0.00)
Blood and cardiovascular system	−0.46 (0.37; 0.22)	0.33 (0.41; 0.43)	0.51 (0.47; 0.29)
Dermatologicals	−0.79 (0.82; 0.33)	1.55⁎ (0.81; 0.05)	−1.00 (1.48; 0.46)
Genito-urinary system and hormones	0.35 (0.75; 0.64)	0.41 (0.74; 0.59)	−1.02 (1.48; 0.45)
Immunomodulators	0.40 (0.41; 0.32)	0.40 (0.42; 0.34)	−2.68⁎⁎ (1.4; 0.01)
Nervous and muscle system	−0.49 (0.44; 0.26)	1.32⁎⁎ (0.46; 0.00)	−1.67⁎ (0.9; 0.04)
Respiratory system	2.39⁎ (1.45; 0.03)	−1.68 (1.46; 0.16)	−0.77 (1.44; 0.57)
Sensory organs	2.21^†^ (1.5; 0.06)	−1.46 (1.49; 0.25)	−0.37 (1.5; 0.80)
Approval pathway:			
Standard	0.61⁎ (0.3; 0.04)	1.26⁎⁎ (0.4; 0.00)	−2.23⁎⁎ (0.39; 0.00)

*Notes:* Statistical significance is indicated as follows: ^†^denotes p < 0.1, ⁎denotes p < 0.05, and ⁎⁎denotes p < 0.01.

By contrast, the proportion of drug indications that did not have any population tested with an appropriate comparator but at least one population with an inappropriate comparator increased from 6/32 (19%) for drug indications approved between 2011 and 2012 to 13/36 (36%) for drug indications approved between 2023 and 2024. The logistic regression estimated 2 times higher odds for drug indications approved in 2023–2024 compared with those approved in 2011–2012. This association was not statistically significant (p-value: 0.13) (Fig. 2, Table 2).

The number of drug indications tested based on a single-arm trial increased from 1/32 (3%) in the approval period 2011–2012 to 9/36 (25%) in the approval period 2023–2024. The logistic regression estimated 7.5 times higher odds (p-value: 0.04) that the drug indication was tested based on a single-arm trial for drug indications approved in 2023–2024 compared with those approved in 2011–2012 (Fig. 2, Table 2).

The results were similar for the sensitivity analysis (eFig. 1). Also, the significant decrease in the proportion of drug indications with at least one population using the appropriate comparator between 2011-2012 and 2023–2024 persisted within both approval pathways when analyzed separately, remaining significant at the 10% level for the standard pathway (regression coefficient −1.11, p-value: 0.06) and at the 5% level for the expedited pathway (regression coefficient −2.61, p-value: 0.03).

The percentage of drug indications with at least one population using an appropriate comparator was significantly higher among drug indications approved through the standard approval pathway (152/246, 62%) than among those with conditional marketing authorization (11/44, 25%), yielding a 37% difference (95% CI: [22%, 51%]). The percentage of drug indications tested based on a single-arm trial was significantly lower for drug indications approved through the standard approval pathway (16/246, 7%) compared to drug indications approved via the conditional marketing authorization pathway (30/44, 68%), corresponding to a −62% difference (95% CI: [–76%, −47%]).

When comparing standard approval with accelerated assessment, the percentage of drug indications with at least one population using an appropriate comparator was 62% (152/246) versus 78% (21/27), which corresponds to a −16% difference (95% CI: [–34%, 2%]). The percentage of drug indications tested based on a single-arm design was 7% (16/246) for drug indications approved through the standard approval pathway versus 7% (2/27) for drug indications granted accelerated assessment, which corresponds to a <0% difference (95% CI: [–11%, 10%]).

## Discussion

In this retrospective cohort study of new drugs approved by the EMA between 2011 and 2024, we found that less than two-thirds of the approved drug indications were tested with an appropriate comparator as determined by the Federal Joint Committee, with a significant decrease over the past decade and a significant increase of single-arm trials. Differences were observed between therapeutic areas.

It is important that comparative data on drugs is generated in a timely manner.^1^, ^8^ An example for the conduction of an efficacy trial with the appropriate comparator is carfilzomib, a drug indicated for the treatment of multiple myeloma. It is notable that the manufacturer conducted a controlled pivotal trial with the appropriate comparator—a combination therapy of lenalidomide and dexamethasone—even though the EMA qualified it as an orphan drug. By contrast, selpercatinib was approved for non-small cell lung cancer. The Federal Joint Committee determined multiple appropriate comparators, nonetheless, the clinical trial was designed as a single-arm trial (eTable 1). When drugs are originally approved without active-comparator trials, such data are unlikely to emerge in the post-marketing period.^8^ Even when post-marketing studies are required by regulatory agencies, they can remain incomplete years after approval.^8^, ^23^, ^24^ Evidence on comparative efficacy guides patients and physicians in their decision making, and also serves reimbursement bodies as a basis for reimbursement decisions and price negotiations.^1^, ^8^

The decline in the proportion of drug indications using an appropriate comparator persisted after adjustment for therapeutic area and approval pathway and within both approval pathways (standard and expedited) when analyzed separately, indicating that it is not explained by the static composition of therapeutic areas or approval pathways across the study period. Other factors that require further study could explain the decreasing use of appropriate comparators. The Federal Joint Committee determines the appropriate comparator during their HTA assessment.^16^ This is usually years after the clinical trials were initiated. Thus, it is possible that the defined appropriate comparator by the Federal Joint Committee was not yet an established therapy at the time of clinical trial initiation. A separate analysis is necessary to assess this and, furthermore, to understand if there were trends over time that are aligned with our study findings. Additionally, manufacturers may have different reasons when deciding which comparator they use for a clinical trial of a new drug. It would be interesting to evaluate the reasons and incentives of the manufacturers for comparator selection and understand if this could explain the study findings.

Some HTA bodies rely on alternative sources of data such as real-world evidence.^25^ However, these sources do not always provide sufficient data both in terms of quantity and quality.^25^, ^26^ It would be beneficial for patients if clinical trials with appropriate comparators were more routinely conducted for new drugs, ideally comparative data should be available at the time of approval.

Second, it would be helpful if drug labels reported information whether trials with appropriate comparators were conducted and which benefits and harms were observed.^8^ Although information on appropriate comparators is available in the decisions of the Federal Joint Committee and the assessment reports of the Institute for Quality and Efficiency in Health Care in Germany, drug labels are the primary tool of regulatory agencies across countries to communicate information about newly approved drugs to patients and physicians.^8^ They guide physicians and patients on safe and effective use of new therapies. Currently, drug labels usually do not include statements about what is or is not known about the relative benefits and harms of new and existing drugs. Without this information, patients and physicians remain largely unaware that most new treatments are not tested against other alternatives.^8^ A national survey in the US showed that almost three-quarters of physicians believed that approvals by the US Food and Drug Administration (FDA) are based on at least comparable effectiveness of a new drug to other approved alternatives.^27^ Improving the content of drug labels could result in a better understanding of available data on benefits and harms, which could improve decision making and subsequent treatment choices of patients and physicians.^28^

Third, reimbursement bodies should consider including the availability of evidence data on comparative efficacy trials with appropriate comparators as a criterion in pricing decisions.^8^ A consequence could be that evidence gaps would result in a price reduction.^29^ Upon successful completion of such comparative efficacy trials, the respective price could be re-negotiated based on the added therapeutic value of the respective drug. In Germany, for example, manufacturers may set the price of a new drug during the first six months after market launch (until 1 January 2023, the period was 12 months).^16^ After this period, the reimbursement price is negotiated based on the added therapeutic value of the new drug compared with the appropriate comparator.^16^ If this comparative data is missing, the price of the drug is set at a lower level.^16^

Also previous studies assessing the comparators of new drugs showed a higher proportion of drugs tested against an appropriate comparator.^6^, ^9^, ^10^, ^11^ However, our study differs from those analyses. Unlike earlier analyses, our cohort included drugs from all therapeutic areas, not only cancer. Also, our cohort covered a more recent time period. Furthermore, we did not determine ourselves whether the appropriate comparator was used but rather relied on the standardized assessments of the Federal Joint Committee, a widely recognized source used by Germany, other countries and researchers.

Our study has limitations. The Federal Joint Committee did not provide an appropriate comparator for all approved drugs (the evaluation was not conducted because the sponsor did not submit any data for the drug indication, the evaluation was still ongoing, orphan drugs without an assessment, reserve antibiotics). Our findings should therefore be interpreted with caution, especially regarding orphan drugs. Also, our evaluation was based on the efficacy trials available at the time of the Federal Joint Committee’s evaluation. We considered both pivotal and supportive trials because some populations were included only in supportive trials. Our results are therefore less conservative than if we had restricted our analysis to pivotal trials. They may also be more generalizable to other jurisdictions as regulatory agencies may differ in which trials they consider pivotal. Clinical trials conducted later or trials for which data were not yet available, were not included. Additionally, the appropriate comparator is usually determined amongst the therapies approved and reimbursed in Germany. A prior study showed that Germany has high and rapid reimbursement rates as drugs are conditionally reimbursed from market launch.^30^ The pool of therapies eligible as comparator is thus relatively broad. Nonetheless, other institutions may rely on different guidelines or references and therefore in some cases determine other drugs as appropriate comparators. Also, the category of inappropriate comparators may range from those, which other institutions would consider as appropriate to those, which are clearly inappropriate. However, we believe that the information from the Federal Joint Committee is reliable, given that it considers both national and international evidence and guidelines and is also used by other countries and scientists.^5^ Finally, there may be several reasons for a sponsor’s selection of comparator and trial design. The absence of a comparator should not necessarily be interpreted as indicating an inappropriate study design, as single-arm trials may be considered sufficient for regulatory decision-making in certain circumstances.^8^ This would require an analysis in a separate study. Less than two-thirds of the approved drug indications were tested with an appropriate comparator, with a significant decrease over the past decade and a significant increase of single-arm trials.

It would be beneficial for patients and physicians if clinical trials with appropriate comparators would be more routinely used for new drug approvals. Second, drug labels could include information whether trials with appropriate comparators were conducted. This would guide physicians and patients in their decision-making. Third, reimbursement bodies could take evidence gaps due to lack of trials with appropriate comparators into account in their price negotiations.

## Contributors

CEGG, SH and KNV designed the study; CEGG, CH, SH and KNV interpreted the results; CEGG and CH collected and verified the data; SH analyzed the data; SH created the figures; CEGG and KNV drafted the manuscript. CEGG, CH and SH accessed and verified the underlying data. All authors had access to all the data, contributed to the revision of the manuscript and approved the final version.

## Data sharing statement

The datasets generated and analyzed for this study are available from the corresponding author after publication upon reasonable request.

## Declaration of interests

CEGG reports a grant from the University of Zurich (Postdoc grant). KNV reports grants from the Swiss National Science Foundation (SNSF) and Swiss Cancer Research Foundation (Krebsforschung Schweiz). KNV is a member of the ESMO Medicines Committee. All other authors declare no competing interests.

## Appendix A Supplementary data

eTable 1 and eFig. 1

## References

[1] Wieseler B., McGauran N., Kaiser T. (2019). New drugs: where did we go wrong and what can we do better?. BMJ.

[2] Sorenson C., Naci H., Cylus J., Mossialos E. (2011). Evidence of comparative efficacy should have a formal role in European drug approvals. BMJ.

[3] Brinkhuis F., Goettsch W.G., Mantel-Teeuwisse A.K., Bloem L.T. (2024). Added benefit and revenues of oncology drugs approved by the European Medicines Agency between 1995 and 2020: retrospective cohort study. BMJ.

[4] Hwang T.J., Ross J.S., Vokinger K.N., Kesselheim A.S. (2020). Association between FDA and EMA expedited approval programs and therapeutic value of new medicines: retrospective cohort study. BMJ.

[5] Vokinger K.N., Glaus C.E.G., Kesselheim A.S., Serra-Burriel M., Ross J.S., Hwang T.J. (2023). Therapeutic value of first versus supplemental indications of drugs in US and Europe (2011-20): retrospective cohort study. BMJ.

[6] Hilal T., Sonbol M.B., Prasad V. (2019). Analysis of control arm quality in randomized clinical trials leading to anticancer drug approval by the US food and drug administration. JAMA Oncol.

[7] Van Luijn J.C.F., Gribnau F.W.J., Leufkens H.G.M. (2007). Availability of comparative trials for the assessment of new medicines in the European Union at the moment of market authorization. Br J Clin Pharmacol.

[8] Naci H., Salcher-Konrad M., Kesselheim A.S. (2020). Generating comparative evidence on new drugs and devices before approval. Lancet.

[9] Hilal T., Mohyudin A., Ayala R., Almader-Douglas D., Leis J.F. (2023). Quality of the control arms in randomized trials of chronic lymphocytic leukemia enrolling in the USA: a systematic review. Eur J Cancer.

[10] Mohyuddin G.R., Koehn K., Sborov D. (2021). Quality of control groups in randomised trials of multiple myeloma enrolling in the USA: a systematic review. Lancet Haematol.

[11] Rossi A., Aimar G., Audisio M. (2023). Analysis of the adequacy of control arms in oncology randomised clinical trials published between 2017 and 2021: a meta-research study. Eur J Cancer.

[12] European Medicines Agency Medicines. https://www.ema.europa.eu/en/medicines.

[13] World Health Organization ATC/DDD index 2025. https://atcddd.fhi.no/atc_ddd_index/.

[14] Institut für Qualität und Wirtschaftlichkeit im Gesundheitswesen (IQWiG) Allgemeine methoden. https://www.iqwig.de/methoden/allgemeine-methoden_v8-0.pdf.

[15] Gemeinsamer Bundesausschuss (G-BA) Frühe Nutzenbewertung wird sachgerecht umgesetzt - G-BA stellt Pressebericht richtig. https://www.g-ba.de/presse/pressemitteilungen-meldungen/403/.

[16] Gemeinsamer Bundesausschuss (G-BA) Verfahrensordnung des Gemeinsamen Bundesausschusses. https://www.g-ba.de/downloads/62-492-4105/VerfO_2025-11-20_iK_2026-04-08.pdf.

[17] Verordnung über die Nutzenbewertung von Arzneimitteln nach § 35a Absatz 1 SGB V für Erstattungsvereinbarungen nach § 130b SGB V, § 6 zweckmäßige Vergleichstherapie. https://www.gesetze-im-internet.de/am-nutzenv/__6.html.

[18] European Medicines Agency Orphan designation. https://www.ema.europa.eu/en/human-regulatory-overview/orphan-designation-overview.

[19] Institut für Qualität und Wirtschaftlichkeit im Gesundheitswesen (IQWiG) (2024). https://www.iqwig.de/presse/pressemitteilungen/pressemitteilungen-detailseite_112512.html#:%7E:text=vorher%20gilt%20ihr%20zusatznutzen%20als,der%20regulären%20nutzenbewertung%20positiv%20bewerten.

[20] Institut für Qualität und Wirtschaftlichkeit im Gesundheitswesen (IQWiG) (2025). https://www.g-ba.de/downloads/17-98-4977/fruehe-nutzenbewertung_gesetzesauftraege.pdf.

[21] Gemeinsamer Bundesausschuss (G-BA) (2025). https://www.g-ba.de/downloads/17-98-4827/2025-11-18_anl2_1_erstellung-einreichung-dossier.pdf.

[22] Gemeinsamer Bundesausschuss (G-BA) Nutzenbewertung von Arzneimitteln - Verfahren nach § 35a SGB V (AMNOG). https://www.g-ba.de/bewertungsverfahren/nutzenbewertung/.

[23] Pease A.M., Krumholz H.M., Downing N.S., Aminawung J.A., Shah N.D., Ross J.S. (2017). Postapproval studies of drugs initially approved by the FDA on the basis of limited evidence: systematic review. BMJ.

[24] Bloem L.T., Mantel-Teeuwisse A.K., Leufkens H.G.M., De Bruin M.L., Klungel O.H., Hoekman J. (2019). Postauthorization changes to specific obligations of conditionally authorized medicines in the European Union: a retrospective cohort Study. Clin Pharm Therap.

[25] Wieseler B., Neyt M., Kaiser T., Hulstaert F., Windeler J. (2023). Replacing RCTs with real world data for regulatory decision making: a self-fulfilling prophecy?. BMJ.

[26] Bartlett V.L., Dhruva S.S., Shah N.D., Ryan P., Ross J.S. (2019). Feasibility of using real-world data to replicate clinical trial evidence. JAMA Netw Open.

[27] Kesselheim A.S., Woloshin S., Eddings W., Franklin J.M., Ross K.M., Schwartz L.M. (2016). Physicians’ Knowledge About FDA Approval Standards and Perceptions of the “Breakthrough Therapy” designation. JAMA.

[28] Schwartz L.M., Woloshin S. (2013). The Drug facts Box: improving the communication of prescription drug information. Proc Natl Acad Sci USA.

[29] Vokinger K.N., Rohner N. (2023). Preisfestsetzung Bei Arzneimitteln - eine Analyse Aktueller Herausforderungen Betreffend Den Auslandpreisvergleich Und Therapeutischen Quervergleich Sowie Entwicklungen von Lösungsvorschlägen. Zeitschrift für Schweizerisches Recht.

[30] Glaus C.E.G., Serra-Burriel M., Dusetzina S.B., Vokinger K.N. (2024). Time from approval to reimbursement of new drugs: a comparative analysis between the United States, England, Germany, France, and Switzerland (2011–2022). Ann Intern Med.

