# Supplementary material for: The use of appropriate, inappropriate and no comparators in efficacy trials for European Medicines Agency-approved drugs 2011–2024: a retrospective cohort study
Source: eClinicalMedicine. 2026 Nov 9;101:104319. doi: 10.1016/j.eclinm.2026.104319 (PMC13770683; doi:10.1016/j.eclinm.2026.104319)
Supplement: Supplementary file 1 [file mmc1.docx]

Supplementary material for:

**The use of appropriate, inappropriate and no comparators in efficacy trials for European Medicines Agency-approved drugs 2011-2024: A retrospective cohort study**

Camille EG Glaus, JD, PhD^1,2^, Cascal Herrle, JD^1,2^, Simon Hediger, PhD^1,2^, Kerstin Noelle Vokinger, MD, JD, PhD^1,2^

**Affiliations:** ^1^Academic Chair for Regulation in Law, Medicine, and Technology, Faculty of Law, University of Zurich, Zurich, Switzerland; ^2^Academic Chair for Regulation in Law, Medicine, and Technology, Department of Health Sciences and Technology, ETH Zurich, Zurich, Switzerland.

**Index**

Supplementary Results

- **eTable 1.** *Examples for appropriate comparators, inappropriate comparators and single-arm trials.*
- **eFigure 1.** Comparators of drug indications overall and over time (sensitivity analysis).

**eTable 1. Examples for appropriate comparators, inappropriate comparators and single-arm trials.**

| **Active substance** | **Indication** | **Populations** | **Trial type** | **Selected comparator (vs appropriate comparator [summary])** |
| --- | --- | --- | --- | --- |
| Tafamidis | Hereditary transthyretin-mediated amyloidosis | Hereditary transthyretin-mediated amyloidosis in adult patients with stage 1 or stage 2 polyneuropathy. | Controlled trial | Appropriate comparator: best supportive care |
| Ertugliflozin/ sitagliptin | Diabetes mellitus 2 | Adults aged 18 years and older with type 2 diabetes mellitus as an adjunct to diet and exercise to improve glycaemic control: when metformin and/or a sulphonylurea (SU) and one of the monocomponents of ertugliflozin/sitagliptin do not provide adequate glycaemic control; in patients already being treated with the combination of ertugliflozin and sitagliptin as separate tablets. | Controlled trial | Inappropriate comparator (appropriate comparator: human insulin in combination with either metformin, empagliflozin or liraglutid or human insulin alone in case of intolerance or contraindication; comparator used: placebo or placebo with metformin) |
| Afatinib | Non-small cell lung cancer (NSCLC) | Untreated patients with ECOG performance status 0 or 1. | Controlled trial | Appropriate comparator: gefitinib or erlotinib or cisplatin in combination with a third-line cytostatic drug (vinorelbine, gemcitabine, docetaxel, paclitaxel, pemetrexed) |
|  |  | Untreated patients with ECOG performance status 2. | No data available | No comparator selected (appropriate comparator: gefitinib or erlotinib or gemcitabine) |
|  |  | Patients previously treated with one or more courses of chemotherapy. | Single-arm | No comparator selected (appropriate comparator: gefitinib or erlotinib) |
| Selpercatinib | Non-small cell lung cancer (NSCLC)  (also approved for medullary thyroid cancer as first indication) | Adults with advanced RET fusion-positive NSCLC for whom systemic therapy is indicated; after first-line therapy with an anti-PD-1/PD-L1 antibody as monotherapy. | Single-arm | No comparator selected (appropriate comparator: Cisplatin in combination with a third-generation cytostatic drug or carboplatin in combination with a third-generation cytostatic drug or carboplatin in combination with paclitaxel or a monotherapy with gemcitabine or vinorelbine) |
|  |  | Adults with advanced RET fusion-positive NSCLC for whom systemic therapy is indicated; after first-line cytotoxic chemotherapy. | Single-arm | No comparator selected (appropriate comparator: Docetaxel or pemetrexed or nivolumab or pembrolizumab or docetaxel in combination with nintedanib) |
|  |  | Adults with advanced RET fusion-positive NSCLC for whom systemic therapy is indicated; after first-line therapy with an anti-PD-1/PD-L1 antibody in combination with platinum-containing chemotherapy or after sequential therapy with an anti-PD-1/PD-L1 antibody and platinum-containing chemotherapy. | Single-arm | No comparator selected (appropriate comparator: Patient-individual therapy taking into account prior therapy and histology; selecting  afatinib, pemetrexed, erlotinib, docetaxel, docetaxel in combination with ramucirumab,  docetaxel in combination with nintedanib and vinorelbine) |

**eFigure 1. Comparators of drug indications overall and over time (sensitivity analysis).**

*Notes:* A drug indication was counted in a category if at least one population within a drug indication fulfilled the respective category (appropriate comparator, inappropriate comparator, single-arm, no data available). Thus, drug indications could be counted in more than one category.
